# Decreased Seasonal Influenza Rates Detected in a Crowdsourced Influenza-Like Illness Surveillance System During the COVID-19 Pandemic: Prospective Cohort Study

**DOI:** 10.2196/40216

**Published:** 2023-12-28

**Authors:** Autumn Gertz, Benjamin Rader, Kara Sewalk, Tanner J Varrelman, Mark Smolinski, John S Brownstein

**Affiliations:** 1 Computational Epidemiology Lab Boston Children's Hospital Boston, MA United States; 2 Department of Epidemiology Boston University School of Public Health Boston, MA United States; 3 Ending Pandemics San Francisco, CA United States; 4 Harvard Medical School Boston, MA United States

**Keywords:** participatory surveillance, influenza, crowdsourced data, disease surveillance, surveillance, COVID-19, respiratory, transmission, detection, survey, sore throat, fever, cough, vaccination, diagnosis, precautions

## Abstract

**Background:**

Seasonal respiratory viruses had lower incidence during their 2019-2020 and 2020-2021 seasons, which overlapped with the COVID-19 pandemic. The widespread implementation of precautionary measures to prevent transmission of SARS-CoV-2 has been seen to also mitigate transmission of seasonal influenza. The COVID-19 pandemic also led to changes in care seeking and access. Participatory surveillance systems have historically captured mild illnesses that are often missed by surveillance systems that rely on encounters with a health care provider for detection.

**Objective:**

This study aimed to assess if a crowdsourced syndromic surveillance system capable of detecting mild influenza-like illness (ILI) also captured the globally observed decrease in ILI in the 2019-2020 and 2020-2021 influenza seasons, concurrent with the COVID-19 pandemic.

**Methods:**

Flu Near You (FNY) is a web-based participatory syndromic surveillance system that allows participants in the United States to report their health information using a brief weekly survey. Reminder emails are sent to registered FNY participants to report on their symptoms and the symptoms of household members. Guest participants may also report. ILI was defined as fever and sore throat or fever and cough. ILI rates were determined as the number of ILI reports over the total number of reports and assessed for the 2016-2017, 2017-2018, 2018-2019, 2019-2020, and 2020-2021 influenza seasons. Baseline season (2016-2017, 2017-2018, and 2018-2019) rates were compared to the 2019-2020 and 2020-2021 influenza seasons. Self-reported influenza diagnosis and vaccination status were captured and assessed as the total number of reported events over the total number of reports submitted. CIs for all proportions were calculated via a 1-sample test of proportions.

**Results:**

ILI was detected in 3.8% (32,239/848,878) of participants in the baseline seasons (2016-2019), 2.58% (7418/287,909) in the 2019-2020 season, and 0.27% (546/201,079) in the 2020-2021 season. Both influenza seasons that overlapped with the COVID-19 pandemic had lower ILI rates than the baseline seasons. ILI decline was observed during the months with widespread implementation of COVID-19 precautions, starting in February 2020. Self-reported influenza diagnoses decreased from early 2020 through the influenza season. Self-reported influenza positivity among ILI cases varied over the observed time period. Self-reported influenza vaccination rates in FNY were high across all observed seasons.

**Conclusions:**

A decrease in ILI was detected in the crowdsourced FNY surveillance system during the 2019-2020 and 2020-2021 influenza seasons, mirroring trends observed in other influenza surveillance systems. Specifically, the months within seasons that overlapped with widespread pandemic precautions showed decreases in ILI and confirmed influenza. Concerns persist regarding respiratory pathogens re-emerging with changes to COVID-19 guidelines. Traditional surveillance is subject to changes in health care behaviors. Systems like FNY are uniquely situated to detect disease across disease severity and care seeking, providing key insights during public health emergencies.

## Introduction

Globally, seasonal respiratory viruses had lower incidence during the 2019-2020 season, which overlapped with the emergence of COVID-19 [1-4]. In the United States, decreases in influenza activity were also observed in 2019-2021 [2,5-8]. A national influenza-like illness (ILI) reference configured using an average of the previous few seasons was established at 2.6%; however, no week within the 2020-2021 season reached an ILI rate above 1.7% [8]. While influenza burden varies seasonally [9], COVID-19 contributed to this widespread decrease [5-7,10].

Many nonpharmaceutical interventions (NPIs) enacted for COVID-19 are also recommended for pandemic influenza [11-13]; therefore, their extensive uptake impacted the cocirculation of influenza. For example, those with mild symptoms for both illnesses are advised to remain home [13]. Traditional disease surveillance may omit these mild illnesses and not capture health care–related changes [14,15]. Crowdsourced estimates can be less susceptible to fluctuations in care seeking resulting from the pandemic [15,16]. Here, we use Flu Near You (FNY), a previously validated crowdsourced influenza surveillance system, to measure seasonal influenza incidence during the COVID-19 pandemic [17].

FNY is uniquely situated to further assess changes in seasonal ILI during the pandemic [14,17,18]. Historically, FNY has provided ILI trend insights that complement traditional surveillance and display compatibility with data from systems like the US Centers for Disease Control and Prevention’s Influenza-like Illness Surveillance Network [17,18]. This study aims to assess whether crowdsourced syndromic surveillance capable of capturing mild influenza also revealed the global decrease in ILI during influenza seasons concurrent with the COVID-19 pandemic.

## Methods

### FNY Participatory Syndromic Surveillance System

Flu Near You (FNY) is a web-based participatory syndromic surveillance system where participants in the United States report their health using a weekly survey. Guest participants may also submit ad hoc reports. Participants are asked if they or household members feel healthy or sick. If sick, participants are prompted with a populated list of symptoms to select (Multimedia Appendix 1). Seasonal influenza vaccination status is also collected. Self-reported influenza diagnosis data were captured starting February 2020.

### Population Sample

Data from 2016-2021 were included, with influenza seasons defined as *Morbidity and Mortality Weekly Report* weeks 40 through 20 (Multimedia Appendix 2, Table S1). Data were included for the entire FNY week that captured the influenza season start and end dates. Weekly reports by both registered and guest participants were included. Registered participants had a single identifier, while guest participants were assigned one per report. FNY used token validation between the front and back ends of the website to reduce the risk of spam reporting. Reports by registered participants were deduplicated by week if multiple reports were captured, with the most recent report included. For demographic analysis, each user was counted once per flu season, and those with missing data were excluded. No further restrictions were applied [17].

### Ethical Considerations

This study of FNY data received approval from the Boston Children’s Hospital Institutional Review Board (IRB-P00023700) and received a waiver of informed consent. FNY participants may report anonymously. Those who opted to provide contact information were deidentified. No compensation was provided to participants.

### Analysis

ILI was defined as fever in addition to sore throat, cough, or both [19]. Influenza diagnosis was classified as self-reported influenza diagnosis (hereafter called self-reported diagnosis). Influenza vaccination rate was defined as the proportion of unique reporters who indicated being vaccinated any time during the influenza season. ILI rates were determined as the number of reports meeting the ILI definition over the total reports during that time. Season and monthly ILI rates were assessed with October and May rates truncated to align with the defined season. CIs were calculated via a 1-sample test of proportions.

Total ILI cases and unique reports from the 2016-2017, 2017-2018, and 2018-2019 influenza seasons were averaged by month and are presented as “baseline seasons.” Demographics across the baseline seasons were aggregated to retain their specific proportions, accounting for participants reporting across seasons. Analyses were conducted with R (version 4.0.4; R Foundation for Statistical Computing).

## Results

### Population

During the baseline influenza seasons, 237,309 unique individuals reported on FNY, while 68,800 individuals reported for the 2019-2020 season and 25,996 in 2020-2021. There were more female respondents to FNY across seasons (Table 1). Higher proportions of older individuals reported across seasons (Table 1). In 2019-2020 and 2020-2021, respondents aged 65 years and older comprised the largest proportions (n=9812, 32.6% and n=8236, 39.9%, respectively) among those who reported age (n=30,139, 43.8% and n=20,650, 79.4%). Those younger than 18 years were the smallest proportion across influenza seasons (Table 1).

**Table 1:** Characteristics of respondents for age and sex.

		Respondents, n (%)		
		Baseline flu seasons (N=274,235^a^)	2019-2020 flu season (n=68,800)	2020-2021 flu season (n=25,996)
Respondents who reported age		85,745 (31.3)	30,139 (43.8)	20,650 (79.4)
**Age group (years)^b^**				
	13-17	6625 (7.7)	2419 (8.0)	1448 (7.0)
	18-34	10,409 (12.1)	3445 (11.4)	2035 (9.9)
	35-44	10,356 (12.1)	3269 (10.9)	1733 (8.4)
	45-54	14,291 (16.7)	4638 (15.4)	2697 (13.0)
	55-64	19,874 (23.2)	6556 (21.8)	4501 (21.8)
	≥65	24,190 (28.2)	9812 (32.6)	8236 (39.9)
Respondents who reported sex		85,741 (31.3)	29,897 (43.5)	20,561 (79.1)
**Sex^c^**				
	Female	52,221 (60.9)	17,980 (56.6)	12,110 (58.9)
	Male	33,520 (39.1)	11,917 (43.4)	8451 (41.1)

^a^Combined demographics from the 2016-2017, 2017-2018, and 2018-2019 influenza seasons.

^b^Percentages reflect the proportion among those who reported age.

^c^Percentages reflect the proportion among those who reported sex.

### ILI Trends

ILI variability was observed across the baseline seasons (Table 2). The characteristics of ILI captured on FNY in the described seasons followed patterns observed in traditional surveillance [9]. The 2016-2017, 2018-2019, and 2019-2020 influenza seasons were classified as moderate, and the 2017-2018 season was severe [9]. On FNY, the observed rate of ILI was 1.51% in 2016-2017, 4.98% in 2017-2018, and 4% in 2018-2019. Across the baseline seasons, the rate of ILI was 3.8%. Both the 2019-2020 and 2020-2021 seasons had lower ILI rates compared to the baseline seasons (Table 2). An ILI rate of 2.55% was observed in 2019-2020 and a rate of only 0.27% was captured in 2020-2021.

**Figure 1:**
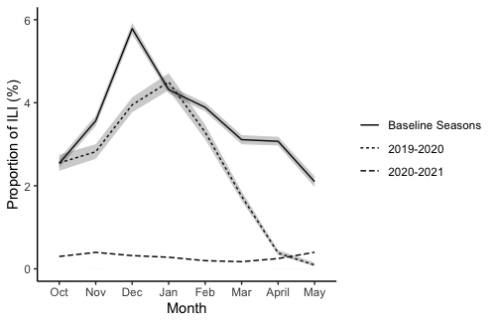
Monthly rates of influenza-like illness (ILI) detected in Flu Near You by influenza season. The 95% CIs are presented with the rates.

Monthly ILI rates displayed expected fluctuations within influenza seasons. The baseline seasons had ILI rates between 2.1% (95% CI 1.89%-2.34%) and 5.79% (95% CI 5.56%-6.02%), with peak activity seen from December to February (Figure 1). ILI activity peaked in January 2020 (4.5%) before moderately declining in February (3.29%) and further declining over the remainder of the season (Figure 1). For the 2020-2021 season, there was no evident peak in ILI (Figure 1).

**Table 2:** Influenza-like-illness (ILI; defined as fever with cough, sore throat, or both) across influenza seasons on Flu Near You.

Influenza season	ILI cases, n	Total reports^a^, n	ILI rate, % (95% CI)	Peak month(s)	Rate during peak month(s), %
2016-2017	3146	207,901	1.51 (1.46-1.57)	Feb	3.82
2017-2018	17,616	353,758	4.98 (4.91-5.05)	Dec	10.17
2018-2019	11,477	287,219	4 (3.92-4.07)	Feb	4.94
2019-2020	7418	287,909	2.55 (2.52-2.64)	Jan	4.5
2020-2021	546	201,079	0.27 (0.25-0.3)	Nov, May	0.4
Baseline seasons^b^	32,239	848,878	3.8 (3.76-3.83)	Dec	5.79

^a^Number of unique weekly reports submitted to Flu Near You.

^b^Combined ILI rate from the 2016-2017, 2017-2018, and 2018-2019 influenza seasons.

### Self-Reported Influenza Diagnosis

Reductions in self-reported influenza diagnosis were detected in FNY (Table 3). Starting at the point of collection (February 2020), the rates of self-reported influenza diagnosis continually decreased (Table 3). February 2020 saw the highest rate of self-reported diagnosed influenza (0.97%) and the highest positivity rate (11.72%) among those tested. The lowest rates of influenza (0.01%) were detected in May 2020 and February and March 2021. From February to May of the 2019-2020 influenza season, the overall influenza rate was 0.21%, and the positivity rate was 7.04%. For the entire 2020-2021 influenza season, there was a lower rate of influenza (0.03%) and a lower positivity rate (1.1%). Reduced reports of influenza-positive ILI were also observed (Table 3).

**Table 3:** Self-reported influenza diagnoses in Flu Near You population.

Time period			Confirmed diagnoses of influenza^a^/total reports, n/N (%)		Positivity^b^ (confirmed diagnoses/total tested for influenza), n/N (%)		Confirmed cases of ILI^c,d^/ILI cases, n/N (%)
**2019-2020**							
	February	389/40,169 (0.97)		389/3318 (11.72)		109/1321 (8.25)	
	March	243/52,499 (0.46)		243/4135 (5.88)		87/919 (9.47)	
	April	18/38,096 (0.05)		18/1197 (1.5)		14/146 (9.59)	
	May	1/16,962 (0.01)		1/517 (0.19)		0/16 (0)	
**2020-2021**							
	October	7/24,812 (0.03)		7/606 (1.16)		5/74 (6.76)	
	November	5/25,023 (0.02)		5/824 (0.61)		3/99 (3.03)	
	December	10/31,181 (0.03)		10/798 (1.25)		0/99 (0)	
	January	13/30,157 (0.04)		13/751 (1.73)		7/84 (8.33)	
	February	4/30,097 (0.01)		4/534 (0.75)		1/59 (1.69)	
	March	4/36,149 (0.01)		4/610 (0.66)		3/62 (4.84)	
	April	4/16,629 (0.02)		4/359 (1.11)		1/41 (2.44)	
	May	6/7031 (0.09)		6/301 (1.99)		2/28 (7.14)	

^a^Self-reported influenza diagnoses among entire reporting population by month.

^b^Self-reported influenza diagnoses among those who reported influenza testing by month.

^c^ILI: influenza-like-illness.

^d^Self-reported influenza diagnoses among those who met the definition of ILI by month.

### Influenza Vaccination

Influenza vaccination rates varied seasonally, with 58.6% (160,610/274,235) of participants being vaccinated in the baseline seasons. Both the 2016-2017 and 2018-2019 seasons had over 80% vaccinated participants (23,632/27,024, 87.4% and 68,588/84,185, 81.5%, respectively). In the 2017-2018 season, which had more guest participants, 42% (68,390/163,026) of participants reported influenza vaccination. The 2019-2020 season had a high vaccination rate of 84% (57,823/68,800). The 2020-2021 season had a lower vaccination rate (66.5%) (17,299/25,996) compared to the previous season.

## Discussion

FNY observed unseasonably low rates of ILI during 2019-2021. Late 2019 had higher rates of ILI than early 2020, mirroring a globally observed reduction in ILI [2,3,5-7,10]. FNY observed ILI decreasing in February 2020, slightly ahead of the nationwide implementation of COVID-19 precautions. This early decrease is likely attributed to high health literacy among FNY participants, as they are probable early adopters of NPIs [17]. For the entirety of the 2020-2021 influenza season, FNY ILI rates were comparable to those outside of the viral season [3,8]. Influenza diagnoses on FNY also decreased from February to May 2020, mirroring ILI trends. This alignment of trends displays the importance of complementing traditional surveillance with participatory systems [20,21], and further suggests that prevention measures for COVID-19 may have secondarily reduced transmission of seasonal influenza.

FNY participants have historically reported influenza vaccination rates around 80% [22], compared to under 50% in the US population [23-25]. Both 2019-2020 and 2020-2021 had higher vaccination rates than the baseline seasons, potentially contributing to low rates of ILI. However, this correlation was impractical to assess due to the high influenza vaccination rates among FNY participants.

This study has limitations worth noting. First, the FNY population is not representative of the US population, limiting the scope of the results [14,15,18]. However, as the FNY data are longitudinal, we are able to compare influenza seasons within this specific population. Second, self-reported data are subject to bias, which can lead to misclassification. Furthermore, ILI is a coarse measurement that may detect other circulating respiratory infections. Therefore, ILI rates may include other seasonal viruses and have inflated baseline levels [1]. Contrarily, COVID-19 may have been captured as ILI. In April and May 2021, there was a delivery issue with FNY reminders, resulting in fewer reports than expected. The minimal increase in detected ILI is likely attributable to less reporting of being healthy, as sick individuals are more likely to report unprompted. Lastly, Outbreaks Near Me [26] was launched in March 2020, contributing to lower reporting on FNY. The low rates of ILI during the 2020-2021 season suggest prevention measures for COVID-19 may have secondarily mitigated transmission of seasonal influenza. However, interpretation of ILI during periods of low circulation should be approached cautiously and may be best studied with the use of zero-inflation models that can appropriately account for zeros in the syndromic surveillance data.

Concerns persist regarding respiratory pathogens re-emerging with COVID-19 guideline changes and declined NPI use [5-7]. The fall 2022 “triple-demic” exemplifies the legitimacy of this concern. Traditional surveillance provides valuable insights but can be subject to delays and changes to the health care system [27]. For example, at-home diagnostics data revealed additional populations traditional surveillance is prone to omit [28]. Crowdsourced surveillance systems like FNY are uniquely situated to detect disease among populations who may be missed by traditional surveillance, thereby providing key insights early in public health emergencies.

## Appendix

Multimedia Appendix 1 Flu near you questionnaire.

Multimedia Appendix 2 Table S1. Influenza season start and end dates.

## Abbreviations

FNY: Flu Near You
ILI: influenza-like illness
NPI: nonpharmaceutical intervention

## Data Availability

The data sets generated during and/or analyzed during this study are available from the corresponding author on reasonable request.
