# Supplementary material for: Decreased Seasonal Influenza Rates Detected in a Crowdsourced Influenza-Like Illness Surveillance System During the COVID-19 Pandemic: Prospective Cohort Study
Source: JMIR Public Health Surveill. 2023 Dec 28;9:e40216. doi: 10.2196/40216 (PMC10784978; doi:10.2196/40216)
Supplement: Multimedia Appendix 1 [file publichealth_v9i1e40216_app1.docx]

## Multimedia Appendix 1

Flu Near You Questionnaire

**[All asked]**

How are you feeling?

- Healthy, thanks!
- Not feeling well

Zip code (free text)

Did you get the flu vaccine on or after July 1st, 20XX?

- Yes
- Not yet
- Not going to

Birth month/year (drop-down)

Sex

- Male
- Female

**[If not feeling well]**

Select all the symptoms you've experienced in the past week.

- Fever
  - Highest recorded temperature
- Fatigue
- Cough
- Body aches
- Runny nose
- Sore throat
- Diarrhea
- Headache
- Shortness of breath
- Nausea
- Rash
- Chills/night sweats

What day did you start feeling ill? (select date)

Did you see a health professional for these symptoms?

- Yes
- No

[If yes to above]

Where did you see the health professional? Select all that apply.

- Doctor's office or HMO
- Urgent care center
- In store clinic
- Emergency room
- Hospitalized overnight
- Virtual visit

What did the health professional say you had?

- Influenza or the flu
- Novel Coronavirus 2019
- Pneumonia
- Bronchitis
- Strep Throat
- An upper Respiratory Infection
- A lower Respiratory Infection
- The common cold
- I do not remember
- Other (specify)

Did the health professional take a nose or throat swab?

- Yes
- No

How many days have you been absent from activities (i.e., work, school, etc.)? (Free text)

Have you traveled outside the United States within the 14 days before symptoms onset?

- Yes
- No

[If yes to above]

What was the last country you traveled to? (Drop-down of all countries)
