# Supplementary material for: Decreased Seasonal Influenza Rates Detected in a Crowdsourced Influenza-Like Illness Surveillance System During the COVID-19 Pandemic: Prospective Cohort Study
Source: JMIR Public Health Surveill. 2023 Dec 28;9:e40216. doi: 10.2196/40216 (PMC10784978; doi:10.2196/40216)
Supplement: Multimedia Appendix 2 [file publichealth_v9i1e40216_app2.docx]

## Supplementary Table 1

S1: Influenza Season Start and End Dates

| **Influenza Season** | **Start Date** | **End date** |
| --- | --- | --- |
|  |  |  |
| 2016-2017 | Oct. 8 | May 20 |
| 2017-2018 | Oct. 7 | May 19 |
| 2018-2019 | Oct. 6 | May 18 |
| 2019-2020 | Oct. 5 | May 16 |
| 2020-2021 | Oct. 3 | May 22 |
